# Modulation of bone remodeling by the gut microbiota: a new therapy for osteoporosis

**DOI:** 10.1038/s41413-023-00264-x

**Published:** 2023-06-09

**Authors:** Zhengtian Lyu, Yongfei Hu, Yuming Guo, Dan Liu

**Affiliations:** State Key Laboratory of Animal Nutrition, College of Animal Science and Technology, China Agricultural University, Beijing, 100193 China

**Keywords:** Bone, Metabolic bone disease, Metabolic disorders

## Abstract

The gut microbiota (GM) plays a crucial role in maintaining the overall health and well-being of the host. Recent studies have demonstrated that the GM may significantly influence bone metabolism and degenerative skeletal diseases, such as osteoporosis (OP). Interventions targeting GM modification, including probiotics or antibiotics, have been found to affect bone remodeling. This review provides a comprehensive summary of recent research on the role of GM in regulating bone remodeling and seeks to elucidate the regulatory mechanism from various perspectives, such as the interaction with the immune system, interplay with estrogen or parathyroid hormone (PTH), the impact of GM metabolites, and the effect of extracellular vesicles (EVs). Moreover, this review explores the potential of probiotics as a therapeutic approach for OP. The insights presented may contribute to the development of innovative GM-targeted therapies for OP.

## Introduction

As a severe skeletal disease, osteoporosis (OP) has garnered considerable attention due to its characteristic symptoms of low bone mass and deterioration of bone microarchitecture, which increases fragility and the risk of fractures.^1^ Individuals over 50 years old, particularly women, are more susceptible to fractures in the hip, vertebral body, and wrist.^2^ Women face a greater risk of developing OP due to bone loss caused by the sharp decline in estrogen levels during menopause.^3^ Moreover, estrogen-independent mechanisms, such as secondary hyperparathyroidism, chronic inflammation, and senility, can also contribute to the development of OP.^4–7^

Bone tissue undergoes continuous renewal through bone remodeling, which is maintained by the equilibrium of osteoclast (OC)-mediated bone resorption and osteoblast (OB)-mediated bone formation.^8^ The development and application of clinical therapeutics for OP, including antiresorptive agents and anabolic agents, primarily depend on the structures and functions of OBs and OCs.^2^ In their review of the currently available drugs for OP, Khosla and Hofbauer noted that some drugs are not recommended for long-term use due to their severe side effects and complications. Consequently, there is an urgent need to develop more effective anti-OP drugs.^9–11^

## The gut microbiota regulates bone remodeling

An imbalance in the gut microbiota (GM), known as dysbiosis, has been observed in individuals with OP.^12–14^ The use of ovariectomized (OVX) mice, a widely utilized animal model in OP studies, has revealed a clear association between GM and bone mass.^14^ Studies involving artificial interference of the GM in animals have demonstrated that the GM acts as a regulator of bone mineral density (BMD).^15–22^ These interventions generally include fecal microbiota transplantation (FMT) in germ-free (GF) mice^15–19^ and antibiotic treatment in conventionally raised mice.^20–22^ Additionally, under physiological conditions, the GM has been shown to influence bone development through “maternal vertical transmission” and “cohabitation transmission”.^23^ In this review, we summarize recent findings on the regulation of bone metabolism by the GM and examine its promising potential as a therapeutic target for OP.

### Evidence from experiments with GF mice

The administration of FMT in GF mice has demonstrated that the GM plays a significant role in regulating bone mass.^15–19^ The initial study that investigated the effect of the GM on bone mass discovered that GF mice exhibited a higher BMD and fewer OCs than wild-type (WT) mice.^15^ Moreover, male GF mice had higher BMD, a higher ratio of bone volume to total volume (BV/TV), and an increased trabecular bone (Tb) number (Tb. N.) in the proximal tibia in comparison to specific pathogen-free (SPF) mice.^16^ Furthermore, the alveolar BMD of GF mice was found to be higher than that of SPF mice.^17^ However, Schwarzer et al. showed that GF mice had shorter femurs and lower cortical bone (Cb) thickness, Cb fraction, and Tb fraction than WT mice.^18^ The variability in these study results could potentially be attributed to the differences in the sex and genetic background of the mice used. The GM exhibited varying regulatory patterns depending on colonization time, as well as the sex and strain of mice. After one month of colonization, female GF mice experienced decreased Tb mass with increased levels of bone absorption marker C-terminal telopeptides of type I collagen (CTX-I) and bone formation marker procollagen type I N-terminal propeptide (P1NP). Furthermore, mice subjected to extended GM colonization had longer femurs than GF mice, despite comparable Tb mass, bone resorption markers, and bone formation markers.^19^

### Evidence from experiments in antibiotic-treated mice

The administration of antibiotics is a model that is widely used to investigate the relationship between the GM and host metabolism.^24^ Weaned female mice exhibited a higher BMD after antibiotic therapy (penicillin, vancomycin, penicillin plus vancomycin, or chlortetracycline; 1 μg·g^−1^ body weight) for three weeks than untreated mice.^21^ Treating parental mice with antibiotics also altered the skeletal structure of their offspring in a gender-specific manner. Compared to the control group, male mice displayed decreased bone mineral content (BMC) and bone area (BA) but unchanged BMD, while female mice exhibited increased BMD with no significant changes in BMC or BA.^20^ One study showed that female mice exposed to therapeutic doses of amoxicillin, tylosin, or a mixture of the two (alternating courses of amoxicillin and tylosin) on days 10–15, 28–31, and 37–40 displayed an increase in BMC and BA across all administrations, with amoxicillin having the most significant effect.^22^ Female mice had increased BV/TV after one month of treatment with broad-spectrum antibiotics (ampicillin, vancomycin, metronidazole, and neomycin). Oral vancomycin alone also increased bone mass, suggesting that gram-positive bacteria may play a substantial role in bone remodeling.^19^ However, administering a four-week course of antibiotics has been shown to disrupt the Tb architecture of the femur.^25^

### Limitations of using GF mice and antibiotic-treated mouse models

It has been shown that GF mice and antibiotic-treated mice serve as effective experimental models for demonstrating the regulation of bone metabolism by the GM. However, they are not considered suitable as screening models for identifying probiotics as potential treatments for OP. The use of GF animals as research models is limited by the absence of a normal GM, which may render the results of such studies potentially inapplicable to individuals or animals with a normal GM. The GM is critical for the postnatal development and maturation of the immune system, which is essential for bone physiology.^26–29^ The underdeveloped lymphoid organs of GF animals, due to a lack of antigenic stimulus, may not reflect real-life scenarios and result in inaccurate findings regarding immunity and related pathways.^30,31^

The use of broad-spectrum antibiotics can have a profound and long-lasting effect on GM composition. While the GM eventually returns to baseline within 8 to 31 months, the composition of bacterial communities often remains altered.^32^ In addition, antibiotics can significantly reduce the abundance of bacteria capable of producing butyrate,^32^ which has been shown to promote bone formation.^33^ Research has also revealed that antibiotic treatment can dramatically reduce the abundance of Bacteroidetes, whose reduction is associated with inflammatory bowel disease (IBD) and type 1 diabetes, both of which involve excessive bone loss.^25,34,35^

In summary, various factors, including the host’s sex and genetics,^16–18^ GM colonization time,^19^ and antibiotic treatment,^21,25^ may influence the composition of the GM. Variations in GM components could partially explain differences observed within the same animal model. Even the same strain of mice from different laboratories may have different GMs.^23^ As a result, it is crucial to focus on the function of individual bacterial strains when evaluating the potential of probiotics as a treatment for OP.

## Regulatory mechanism of bone remodeling by the GM

**Fig. 1:**
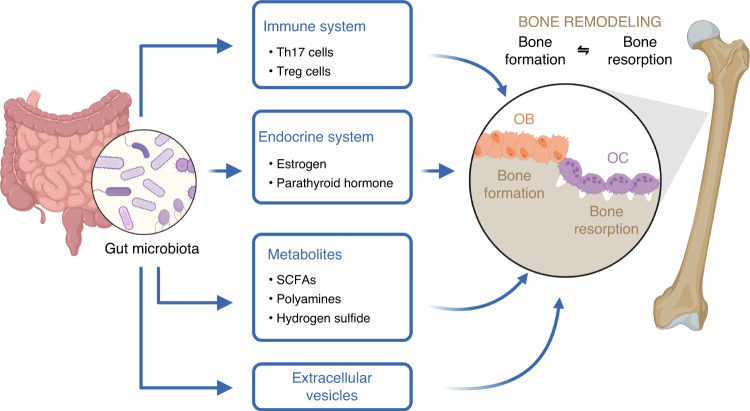
Regulation mechanisms of bone metabolism by gut microbiota. GM indirectly impacts bone metabolism by interacting with the immune system, maintaining a balance between Th17 cells and Treg cells. Additionally, GM or its metabolites have been demonstrated to be pivotal in regulating bone remodeling through their effect on estrogen and PTH. Moreover, GM regulates bone remodeling directly through its microbial metabolites, such as SCFAs, polyamines, and H_2_S, as well as EVs. EVs Extracellular vesicles, H_2_S Hydrogen sulfide, SCFAs Short-chain fatty acids, Th17 cells T helper cells 17, Treg cells T Regulatory cells. Created with BioRender.com

As illustrated in Fig. 1, the regulatory mechanism of bone metabolism by the GM includes direct and indirect effects. Directly, GM influences bone remodeling through the release of extracellular vesicles (EVs) or microbial metabolites such as short-chain fatty acids (SCFAs), polyamines, and hydrogen sulfide (H_2_S). Indirectly, the GM regulates bone remodeling by its interaction with immune cells, such as T helper cells 17 (Th17 cells) and T regulatory cells (Treg cells), or hormones such as estrogen and parathyroid hormone (PTH). This section delves into the various mechanisms through which the GM affects bone remodeling.

### The GM regulates bone remodeling through the immune system

The intestine is the largest lymphatic organ and is host to a diverse array of microorganisms. The instrumental role of the GM in immune maturation, homeostasis, and inflammatory diseases has been widely recognized.^36^ A dysbiotic GM is associated with increased intestinal permeability, characterized by decreased expression of intestinal tight junction proteins and leading to bacterial translocation, chronic inflammation, and the migration of inflammatory cells.^25,30^ This persistence of intestinal inflammation often results in chronic inflammatory diseases that are associated with bone destruction, even if the inflammatory site is not located in the bone, as seen in IBD or Crohn’s disease (CD).^37^ Significant bone loss has been observed in various models of intestinal inflammation, including in dextran sulfate sodium (DSS)-induced chemical injury, adoptive T-cell transfer of colitis, and *Salmonella enterica* infection.^38^ Several chemokines and inflammatory cytokines in the femur, including granulocyte colony-stimulating factor (G-CSF), tumor necrosis factor-α (TNF-α), interleukin (IL)-12p40, MCP-1/CCL-2, RANTES/CCL-5, and keratinocyte-derived chemokine/CXCL1, increased dramatically across multiple colitis models, resulting in the expansion of osteoclast precursor cells (pre-OC).^38^ The receptor activator of nuclear factor kappa-B ligand (RANKL), due to its expression in both activated T cells and mesenchymal lineage cells, is considered an irreplaceable molecule that bridges the immune system and skeletal system.^39–43^ The term “osteoimmunology” highlights the reciprocal interactions between the skeletal and immune systems.^44,45^

#### T helper 17 cells

**Fig. 2:**
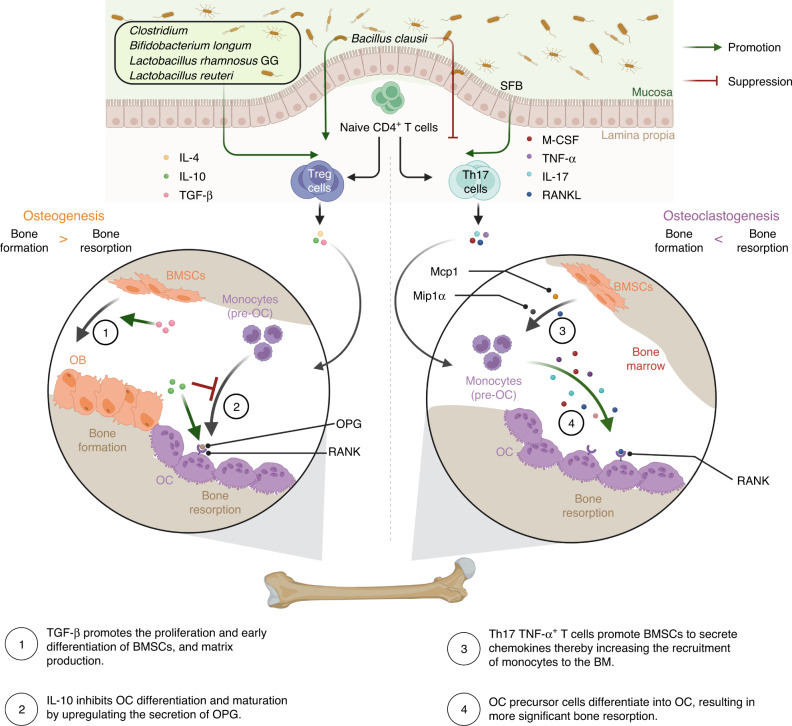
Gut microbiota regulates bone metabolism through the immune system. Th17 and Treg cells exhibit opposite effects on bone remodeling. GM influences bone remodeling by modulating the balance between Th17 and Treg cells. Th17 cells promote OC differentiation, leading to heightened bone resorption. Conversely, Treg cells inhibit osteoclastogenesis and increase bone formation by secreting anti-inflammatory cytokines such as IL-4, IL-10, and TGF-β. BM Bone marrow, BMSCs Bone marrow mesenchymal stem cells, IL Interleukin, M-CSF Macrophage colony-stimulating factor, OB Osteoblasts, OC Osteoclasts, OPG Osteoprotegerin, RANK Receptor activator of nuclear factor kappa-B, RANKL Receptor activator of nuclear factor kappa-B ligand, SFB Segmented filamentous bacteria, TGF-β Transforming growth factor-β, Th17 cells T helper cells 17, TNF-α Tumor necrosis factor-α, Treg cells T Regulatory cells. Created with BioRender.com

Early studies indicated that activated T cells were an essential source of RANKL.^46^ Subsequent research, however, revealed that not all activated T cells have the capability to stimulate OC differentiation. Only Th17 TNF-α^+^ cells selectively express macrophage colony-stimulating factor (M-CSF) and RANKL, but not interferon (IFN)-γ, which acts as an inhibitor of OC differentiation.^35,46^ The mechanism by which Th17 cells regulate bone metabolism is depicted in Fig. 2. Bone marrow (BM) Th17 TNF-α^+^ cells not only promote OC differentiation in the absence of exogenous osteoclastogenic factors but also stimulate bone marrow mesenchymal stem cells (BMSCs) to secrete chemokines (Mcp1, Mip1α, and RANKL) and recruit inflammatory monocytes (pre-OC) to the BM, resulting in increased bone resorption.^35^ Certain bacteria, such as segmented filamentous bacteria (SFB), *Bifidobacterium adolescentis*, and *Eggerthella lenta*, have been reported to expand Th17 cells.^47–49^ As a gram-positive commensal bacterium, SFB stimulates an increase in Th17 cells and the secretion of IL-17 in the gut.^47,50–52^ SFB has been demonstrated to disrupt BMD through maternal vertical transmission and cohabitation transmission.^23,53,54^

Th17 cells play a vital role in OVX-induced bone loss.^30,55^ These cells stimulate osteoclastogenesis and bone resorption through the elevated production of IL-17, RANKL, and TNF-α.^56^ Premenopausal women who underwent OVX have higher numbers of activated circulating CD3^+^ CD69^+^ T cells and CD3^+^ TNF^+^ cells in their peripheral blood than healthy individuals.^57^ An increase in Th17 TNF-α^+^ T-cell-mediated TNF-α is considered the predominant factor involved in PTH-induced bone loss.^5^ Th17 cells, which contain the vβ14^+^ chain on their T-cell receptor, are typically only found in the lamina propria under noninflammatory conditions.^58^ However, the administration of continuous parathyroid hormone (cPTH) has been shown to increase the number of vβ14^+^ Th17 cells in the BM, which depends on the presence of SFB.^5^ TNF increases Th17 cells (CD4^+^ IL-17A^+^ T cells) in the gut in concert with SFB and guides the migration of intestinal Th17 cells to BM through the upregulation of CCL20.^5^ A recent study found that aging leads to an accumulation of immune cells (including neutrophils, monocytes-macrophages, and M1-like macrophages) in the BM of 18-month-old rats.^59^ The abundance of grancalcin (GCA) secreted by neutrophils and monocytes-macrophages in the BM disrupts skeletal microarchitecture by promoting adipogenic differentiation and suppressing bone turnover at the expense of ossification, which are well-known characteristics of senescent BMSCs.^59,60^

The following treatment options can be considered based on the above research: 1. Inhibition of the transportation of immune cells to the BM. FTY720, an inhibitor of sphingosine 1 phosphate (S1P) receptor-1, can arrest lymphocyte transportation from Peyer’s patches (PPs) and mesenteric lymph nodes without affecting lymphocyte function. FTY720 has been shown to reduce the number of Th17 cells and Vβ14^+^ Th17 cells in the BM.^5^ 2. Transport BM immune cells back to the intestinal tract. Oral administration of synthetic retinoid AM80 upregulates the expression of gut-homing molecule α4β7 on T cells, transferring T follicular helper cells (CD19^−^ CD4^+^ CXCR^+^ PD-1^+^ T cells) from the inflammation site to PPs and thereby reducing the inflammatory response.^61^ Increasing the migration of T cells from bone to intestine-related lymphoid tissues theoretically minimizes T-cell-derived RANKL. However, some drugs used to treat IBD target anti-α4β7 integrin (vedolizumab), which acts therapeutically by blocking the gut homing of T lymphocytes.^62–64^ Consequently, methods that address both conditions (OP or IBD) are urgently needed. 3. Neutralize inflammatory factors with antibodies. A neutralizing IL-17A antibody can inhibit RANKL expression and bone loss in OVX mice. Deficiency of IL-17RA or Act1, an IL-17RA-interacting protein, protects mice from OVX-induced bone loss.^65^

#### T Regulatory cells

Contrary to the impact of Th17 cells, Treg cells, which are CD4^+^ T cells with immunosuppressive functions, have a beneficial impact on bone remodeling.^48^ Certain microbes regulate bone remodeling by altering the balance between Th17 cells and Treg cells. For example, oral administration of *Bacillus clausii* has been shown to significantly increase the population of CD4^+^ Foxp3^+^ Treg cells and decrease the proportion of CD4^+^ Rorγt^+^ Th17 cells in the BM and spleen, thereby preventing OVX-induced bone loss.^66^ Clostridia have demonstrated considerable benefits in Treg cell populations.^67–70^ Furthermore, specific bacterial species such as *Lactobacillus rhamnosus* GG (LGG), *Lactobacillus reuteri* (*L. reuteri*), *Bifidobacterium breve* (*B. breve*) AH1205*, Bifidobacterium longum* (*B. longum*) AH1206 and probiotic mixture VSL#3 (a mixture of *B. breve, B. longum, Bifidobacterium infantis, Lactobacillus acidophilus, Lactobacillus plantarum* (*L. plantarum*)*, Lactobacillus paracasei, Lactobacillus bulgaricus*, and *Streptococcus thermophilus*) have the potential to impact both the abundance and functionality of Treg cells.^33,71–74^

The mechanism by which Treg cells regulate bone metabolism is illustrated in Fig. 2. Treg cells inhibit osteoclastogenesis and promote bone formation by secreting anti-inflammatory cytokines such as IL-4, IL-10, and transforming growth factor-β (TGF-β).^44,75,76^ IL-10, an inhibitory cytokine, helps to downregulate the expression of RANKL and M-CSF and enhances the secretion of osteoprotegerin (OPG), thereby inhibiting OC differentiation and maturation.^77^ TGF-β plays a critical role in regulating various stages of OB differentiation, promoting the proliferation and early differentiation of OB progenitor cells and stimulating matrix production but inhibiting later differentiation and matrix mineralization.^78^ A mixture of Clostridia strains belonging to clusters IV, XIVa, and XVIII can activate intestinal epithelial cells to produce TGF-β in the colon.^69^ Furthermore, Treg cells have the capacity to stimulate CD8^+^ T cells to secrete the Wnt ligand Wnt10b, which stimulates bone formation by activating Wnt signaling in OBs.^33^ The administration of LGG did not result in any increase in BV and bone formation in mice depleted of Treg cells through anti-CD25 antibody treatment, highlighting the crucial role of Treg cells in the bone anabolic activity of LGG.^33^

### The role of the GM in estrogen regulation of bone metabolism

**Fig. 3:**
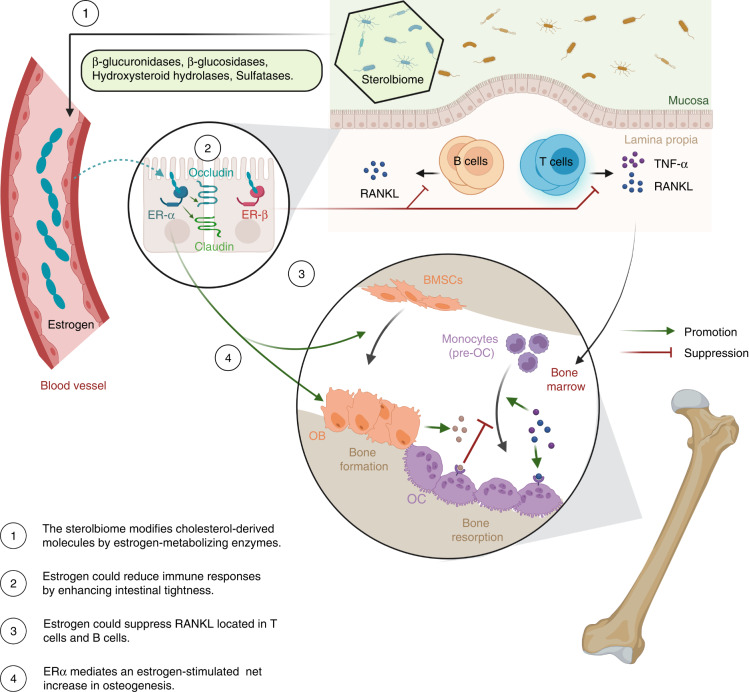
The role of gut microbiota in estrogen regulation of bone metabolism. Estrogen has an irreplaceable role in osteogenesis and osteoclastogenesis. Some “sterolbiome”, which could modify these cholesterol-derived molecules, regulates bone remodeling by interaction with estrogen. BMSCs Bone marrow mesenchymal stem cells, ER Estrogen receptor, OB Osteoblasts, OC Osteoclasts, OPG Osteoprotegerin, pre-OC Osteoclast precursor cells, RANK Receptor activator of nuclear factor kappa-B, RANKL Receptor activator of nuclear factor kappa-B ligand, TNF-α Tumor necrosis factor-α. Created with BioRender.com

Accumulated research has revealed the irreplaceable role of estrogen in osteogenesis and osteoclastogenesis.^46,79–83^ As illustrated in Fig. 3, estrogen receptor α (ERα) mediates an estrogen-stimulated net increase in bone formation.^84,85^ A lack of ERα results in reduced femur length in female adult mice.^86^ The regulation of bone metabolism by estrogen also has close ties to the immune system, as it protects bone mass through the downregulation of immune responses and modulation of the balance between OB and OC.^87^ Estrogen suppresses RANKL located in CD3^+^ T cells and CD20^+^ B cells^88^ and elevates the production of OPG in osteoblastic cells.^89,90^ Bone loss resulting from decreased estrogen is due to the T-cell-mediated increase in TNF-α, which indirectly enhances OC differentiation.^91–94^ The activation of nuclear factor kappa-light-chain-enhancer of activated B cells (NF-κB) in response to sex steroid deficiency promotes bone resorption and impedes bone formation.^90^

The role of the GM in regulating estrogen metabolism is attracting growing interest. The GM directly contributes to the control of host sex steroid levels.^95^ The term “sterolbiome” refers to the collection of gut microbes that modify these cholesterol-derived molecules.^96^ The most critical estrogen-metabolizing enzymes encoded by the sterolbiome are β-glucuronidases, β-glucosidases, hydroxysteroid hydrolases, and sulfatases, which deconjugate estrogens to enhance intestinal reuptake.^97,98^ These enzymes and their activities are well represented in the human GM and are modulated by diet and bacterial density, leading to changes in local and systemic estrogen levels.^99,100^ Li et al. reported that sex steroid deficiency-associated bone loss was dependent on the GM. Estrogen deficiency induced by leuprolide, a gonadotrophin-releasing hormone agonist, was insufficient to increase bone resorption and Tb loss in GF mice. Mechanistically, estrogen deficiency led to decreased expression of intestinal tight junction proteins, increased intestinal permeability, and elevated serum endotoxin levels; however, these phenotypes were unaffected in GF mice. Only conventionally raised mice showed increased expression of osteoclastogenic cytokines in both the BM and small intestine after estrogen deprivation,^30^ thus indicating that the bone loss associated with estrogen deficiency is linked to GM-driven inflammatory signaling.

### The role of the GM in parathyroid hormone regulation of bone metabolism

**Fig. 4:**
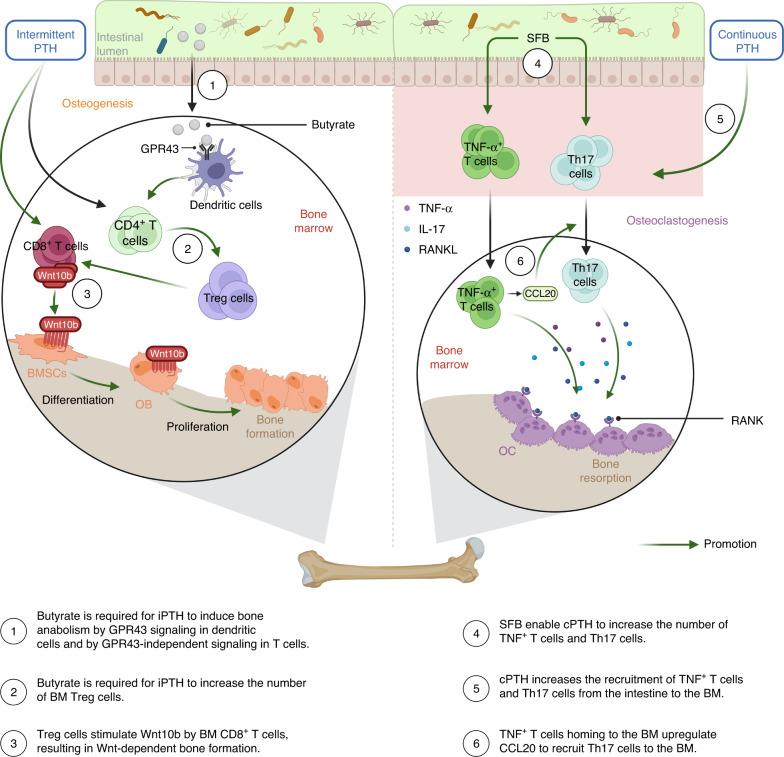
The role of gut microbiota in PTH regulation of bone metabolism. PTH plays a vital role in postnatal skeletal development. SFB enhances the inflammatory responses by interacting with cPTH in the gut and BM, leading to bone loss. However, the GM metabolite butyrate is essential for iPTH to stimulate bone formation. BM: Bone marrow, BMSCs Bone marrow mesenchymal stem cells, cPTH Continuous parathyroid hormone, iPTH Intermittent parathyroid hormone, GPR G-protein-coupled receptor, IL Interleukin, OB Osteoblasts, OC Osteoclasts, PTH Parathyroid hormone, RANK Receptor activator of nuclear factor kappa-B, RANKL Receptor activator of nuclear factor kappa-B ligand, SFB Segmented filamentous bacteria, Th17 cells T helper cells 17, TNF-α Tumor necrosis factor-α, Treg cells T Regulatory cells. Created with BioRender.com

As a pivotal hormone regulating calcium balance, PTH plays a critical role in postnatal skeletal development.^101^ The effect of PTH on bone remodeling, as depicted in Fig. 4, is contingent upon the pattern of exposure of target cells to PTH - whether it is continuous or intermittent.

#### Continuous parathyroid hormone

Primary hyperparathyroidism, a condition characterized by chronic continuous overproduction of PTH by the parathyroid glands,^102^ can be modeled in animals through cPTH infusion.^103^ This condition, which is a common cause of OP and fractures,^104,105^ is characterized by the critical role of osteocyte-derived RANKL and T-cell-derived IL-17A in promoting bone catabolism.^106^ However, cPTH was unable to induce bone loss in either antibiotic-treated or GF mice. The presence of SFB in the GM enabled cPTH to increase the number of intestinal TNF^+^ T cells and Th17 cells, as well as the recruitment of these cells from the intestine to the BM. The TNF^+^ T cells in the BM upregulated CCL20, a chemoattractant that facilitated the recruitment of Th17 cells from the intestine to the BM.^5^

#### Intermittent parathyroid hormone

Maximizing the bone anabolic effects of PTH can be achieved by administering daily injections of intermittent PTH (iPTH) in young mice.^107,108^ This intervention results in a marked increase in both BV and strength by stimulating bone formation.^109,110^ The activation of Wnt signaling in osteoblastic cells drives these effects, which are characterized by enhanced OB formation, extended lifespan, and reactivation of bone lining cells.^110,111^

A recent study revealed that the permissive levels of butyrate produced by the GM are essential for iPTH to induce bone anabolism in mice.^54^ In the absence of the GM, the anabolic effects of iPTH and antibiotic treatment resulted in no increase in Treg cells in the gut and BM. However, the administration of butyrate restored the bone anabolic activity of iPTH and increased the number of Treg cells. By binding to G-protein-coupled receptor 43 (GPR43) located in dendritic cells, butyrate stimulates Treg differentiation, which then triggers the expression of the Wnt ligand Wnt10b in BM CD8^+^ T cells and activates Wnt-dependent bone formation.^54^

### The GM regulates bone physiology via metabolites

The GM exerts positive effects on distal organs through the production of secondary metabolites, which serve as essential regulators of anatomically distant organs.^112^ These metabolites are now commonly referred to as “postbiotics”.^113^

#### Short-chain fatty acids (SCFAs)

Vegetable fiber is a dietary element that plays an important role in human health and microbial composition.^112^ SCFAs, including acetate, propionate, and butyrate, are produced through microbial fermentation of nondigestible dietary fiber.^114^ While acetate can be produced by various bacterial species, the production pathways for propionate and butyrate are more limited and involve specific bacterial strains.^115^ *Akkermansia muciniphila* (*A. muciniphila*) generates propionate by digesting intestinal mucin.^116^ Additionally, several bacterial species, including *Eubacterium dolichum*, *Ruminococcus bromii* (*R. bromii*), *Bacteroides eggerthii*, *Bacteroides fragilis*, and *Veillonella parvula*, have been linked to propionate production in the intestine.^117^ The majority of butyrate-producing bacteria in the human gut belong to the families Clostridiaceae, Eubacteriaceae, Lachnospiraceae, and Ruminococcaceae.^118^ Representative species of these families, such as *Faecalibacterium prausnitzii*, *Eubacterium rectale*, *Eubacterium hallii*, *R. bromii*, and *Clostridium butyricum*, have been identified as the key butyrate producers.^119–121^

Acetate, propionate, and butyrate significantly increased bone mass through distinct mechanisms. Acetate inhibits OC numbers in a T-cell- and B-cell-dependent manner,^120^ while propionate and butyrate effectively prevent OVX-induced bone loss by decreasing OC number and serum CTX-I levels. However, acetate did not show any protection against OVX-induced bone loss.^120^ Additionally, acetate, propionate, and butyrate also suppressed OC differentiation in vitro,^120^ which may be related to their inhibition of histone deacetylase activity.^112,122–124^ Butyrate inhibits osteoclastogenesis by suppressing c-Fos, tumor necrosis factor receptor associated Factor 6 (TRAF6), and nuclear factor of activated T cells cytoplasmic 1 (NFATc1) expression.^120,125,126^

Studies of SCFAs in bone formation have mainly focused on butyrate. After treatment with butyrate, mice exhibited a substantial increase in BV/TV, serum osteocalcin (OCN), and mineral apposition rate (MAR) after two and four weeks compared to those treated with a control vehicle.^33^ The increase in femoral BV/TV by butyrate disappeared following inhibition of Treg cells with a CD25 antibody, demonstrating that the capacity of butyrate to stimulate bone formation was Treg-dependent.^33^ While Lucas et al. reported that butyrate improves bone mass by inhibiting osteoclastogenesis instead of promoting bone formation,^120^ early studies in the 2000s showed that butyrate promotes osteogenic differentiation of human BMSCs.^127,128^ These studies demonstrate butyrate’s ability to promote bone formation, although the mechanisms appear contradictory. While studies have shown that the administration of butyrate increased BMSC proliferation in WT mice but not in mice treated with CD25 antibodies,^33^ it remains unclear whether CD25 antibodies affect OB mineralization by butyrate in vitro. Although initial studies have explored the mechanism of butyrate’s promotion of bone formation in BMSCs, further research is necessary using various cell lines or animal models to provide a complete understanding. Studies of acetate and propionate on OB are limited. Acetate was recently reported to increase the differentiation of aging BMSCs by restoring cytosolic acetyl-CoA levels and remodeling the chromatin landscape.^60^

The effective regulation of SCFAs in bone remodeling highlights their potential role in novel therapeutic strategies in OP treatment. Researchers have reported that a vegetarian diet or a Mediterranean diet is beneficial to bone health.^129,130^ Maximizing SCFA levels by supplementing a diet rich in vegetable fiber or SCFA-producing bacteria may provide an approach to prevent against OP.

#### Polyamines

Ubiquitous in all organisms, polyamines are naturally occurring organic polycations derived from amino acids and are involved in various biological processes, such as proliferation, differentiation, and apoptosis.^131^ With a fast plasma turnover and the ability to quickly reach target tissues,^132^ the majority of the host polyamine pool is comprised of polyamines originating from the GM.^133^ Gut microbes primarily produce polyamines through the transamination of ingested amino acids, particularly arginine, by catalytic enzymes.^134,135^ The decrease in total bacteria by antibiotic administration resulted in the depletion of spermine levels in the intestine.^133^ Additionally, an increased abundance of *A. muciniphila* may be associated with polyamine biosynthesis.^8^

Polyamines have been shown to promote osteogenic differentiation of goat adipose tissue‐derived mesenchymal stem cells (ADSCs) and mouse BMSCs.^8,136,137^ Lee et al. demonstrated that exogenous polyamines regulated osteogenic and adipogenic differentiation in a reciprocal manner,^131^ upregulating osteogenic gene expression (including runt-related transcription Factor 2 (RUNX2), alkaline phosphatase (ALP), osteopontin, and OCN) and downregulating adipogenic gene expression (such as peroxisome proliferator-activated receptor (PPAR-γ)), thereby reducing fat accumulation and promoting extracellular matrix mineralization and osteogenesis in human BMSCs.^131^ Additionally, polyamines are known inhibitors of osteoclastogenesis, with oral administration of spermidine or spermine directly preventing an increase in the OC surface/bone surface ratio and a decrease in the BV in OVX mice.^138^ Either spermine or spermidine could reduce the number of multinucleated tartrate-resistant acid phosphatase (TRAP)^+^ cells in a concentration-dependent manner in vitro.^138^ More recently, daily oral supplementation of a diet containing polyamine-rich yeast was found to inhibit osteoclastic activation in OVX mice.^139^ However, inhibition of polyamine biosynthesis in vivo has limited the beneficial effects of spermine and spermidine on bone strength.^8^

Excessive spermidine concentrations were associated with an increased risk of osteoporotic fracture.^140^ Spermine synthase is responsible for synthesizing spermine from spermidine. Deficiency of spermine synthase causes excessive spermidine accumulation and a lack of spermine.^141^ Patients with Snyder-Robinson syndrome, a syndrome caused by loss-of-function mutations of the spermine synthase gene, exhibit severe OP and kyphoscoliosis and have BMSCs with impaired capacities for osteogenic differentiation and mineralization.^142^

#### Hydrogen sulfide

As a vital endogenous gasotransmitter, H_2_S is produced through endogenous cysteine catabolism and sulfate-reducing bacteria (SRB).^112,143–145^ Germ-free mice have been found to have significantly lower levels of free H_2_S in the cecum and colon than conventional mice.^143^ Cysteine catabolic bacteria, including *Fusobacterium*, *Clostridium, Escherichia*, *Salmonella*, *Klebsiella*, *Streptococcus*, *Desulfovibrio*, and *Enterobacter*, convert cysteine to H_2_S, pyruvate, and ammonia through the action of cysteine desulfhydrase. Sulfate-reducing bacteria, including *Desulfovibrio*, *Desulfobacter*, *Desulfobulbus*, and *Desulfotomaculum*, are responsible for producing H_2_S, with *Desulfovibrio* being the dominant genus, and *Desulfovibrio piger* and *Desulfovibrio desulfuricans* being the dominant species.^145^ H_2_S serves as a critical metabolite for GM-mediated bone remodeling.

H_2_S has been implicated in bone formation and postnatal skeletal development, with the ability to maintain the self-renewal and osteogenic differentiation of BMSCs through the Wnt/β-catenin signaling pathway.^146^ Sodium hydrosulfide (NaHS), a common H_2_S donor, has been shown to decrease the RANKL/OPG mRNA ratio in human BMSCs.^147^ In pre-OCs, NaHS inhibited OC differentiation by reducing intracellular reactive oxygen species (ROS) levels and triggering nuclear factor erythroid 2-related Factor 2 (NRF2)-dependent antioxidant responses.^147^ In pathologic bone loss, OVX decreased the concentration of serum H_2_S and two key H_2_S-generating enzymes (cystathione β-synthase and cystathione γ-lyase) in the BM.^148^ H_2_S deficiency in cystathione β-synthase knockout mice leads to a consistently osteoporotic phenotype^149^ with impaired BMSCs and impaired bone formation.^146^ Restoration of H_2_S levels by the H_2_S donor GYY4317 reversed the osteopenia phenotype and osteogenic deficiency of BMSCs.^146^ The administration of GYY4137 increases serum H_2_S levels and bone formation by activating Wnt signaling via increased Wnt10b production and prevents the loss of Tb.^148^

### Gut microbe-derived extracellular vesicles

Extracellular vesicles (EVs), which bacteria release as a means of interspecies communication, are spherical lipid bilayer nanostructures with diameters ranging from 10 to 400 nm. These nanostructures are comprised of various components, including bioactive proteins, lipids, nucleic acids, and virulence factors.^150^ Their unique nanoscale structure ensures the long-distance transport of EVs and their interior molecules throughout the intracellular compartments in a concentrated, protected, and targeted manner.^151^ Moreover, EVs released by different microbes exhibit different characteristics, such as different morphology, composition, and biogenesis. Additionally, environmental conditions can impact the protein profiles of EVs.^152^

Tong et al. isolated EVs from LGG that had a diameter of 161.9 ± 54.8 nm and expressed the exosomal protein TSG101. Oral administration of LGG-released EVs effectively ameliorated DSS-induced colitis by inhibiting Toll-like receptor (TLR) 4/NF-κB/nod-like receptor family 3 (NLRP3) axis activation. Treatment with LGG-EVs also reshaped the GM in colitis mice, with characteristic beneficial bacteria such as *A. muciniphila* and *Bifidobacterium animalis* clustering in the LGG-EV group.^153^ Administration of *L. reuteri*-EVs attenuated LPS-induced inflammation in broilers, thereby improving growth performance, reducing mortality, and reducing intestinal injury.^154^ Administration of EVs released from *Lactobacillus sakei* NBRC15893 was reported to enhance immunoglobulin A production by activating host TLR2 signaling.^155^ In addition, EVs derived from *A. muciniphila* were effective in protecting against DSS-induced colitis.^156^ Administration of *A. muciniphila*-released EVs was also found to regulate lipid metabolism and inhibit inflammation in the colon, adipose tissue, and liver, thereby ameliorating high-fat diet-induced obesity.^157,158^ The “gut-bone axis” has received limited attention regarding research on EVs, with only one study demonstrating that the protective effect of *A. muciniphila* on bone is mediated by the secretion of EVs. These nanovesicles enter and accumulate in bone tissues, inhibiting osteoclastogenesis and promoting osteogenesis.^159^

Due to the potential dangers of genetically modified organisms in clinical settings, EVs are considered an alternative to probiotics in some immunocompromised individuals.^152^ Chronic diseases such as inflammation or obesity are recognized as being closely related to bone metabolism. Further investigation is required to clarify the mechanism of the GM in the “gut-bone axis” from the perspective of EVs. However, producing sufficient quantities of EVs with high purity and reproducibility remains a critical challenge for subsequent studies.^160^

## The prospect of probiotics in osteoporosis therapy

Fecal microbiota transplantation may not be an effective option for the treatment of OP due to the presence of harmful bacteria within the transplant material. Studies have shown that the therapeutic effects of FMT for IBD are also inconsistent.^36^ Therefore, it is essential to thoroughly study the function of bacterial species and evaluate their safety. Probiotics are defined as viable microorganisms that provide a health benefit when administered in adequate quantities.^161^ As a potential new therapy for OP, probiotics are gaining attention. The effects and involved mechanisms of probiotics on bone remodeling are summarized in Table 1.

**Table 1:** The effects and underlying mechanisms of probiotics on bone remodeling in animal models and population-based studies

Genus	Species	Research models or population	Effects	Mechanisms	References
*Lactobacillus*	*L. reuteri* ATCC PTA 6475	OVX Balb/c mice	Femur and vertebrae BV/TV, BMD, BMC, Tb. N. ↑ , Tb spacing ↓	TRAP5 and RANKL mRNA expression ↓ , BM CD4^+^ T cells, OC differentiation in vitro ↓	^173^
	*L. reuteri* ATCC PTA 6475	Type 1 diabetic C57BL/6 mice	Tb. BV/TV, Tb. N., Tb. Th., BMD, BMC ↑ , Tb spacing ↓	MAR, OB surface, serum OCN, Wnt10b expression and Wnt10b^+^ cells in gut and bone ↑ , BM adipocyte area and size ↓	^34^
	*L. reuteri* ATCC PTA 6475	BALB/c mice with OP	Femur and vertebrae BV/TV, BMD, BMC, Tb. N., MAR, serum OCN ↑ , Tb spacing, serum TRAP 5b ↓	Colon permeability, colon TNF-α/IL-10 ratio ↓	^25^
	*L. reuteri* ATCC PTA 6475	Rag knockout C57BL/6 mice (deficient in mature T- and B- cells)	Femur BV/TV, BMC, Tb. Th. in WT mice ↑	IL-10, IFN-γ, TGF-β, osterix gene expression ↑ , *L. reuteri* requires lymphocytes to exert beneficial effects on bone.	^181^
	*L. rhamnosus* GG	OP C57BL/6 J mice induced by leuprolide administration	Spine and femur BV/TV, serum OCN ↑ , serum CTX ↓	Intestinal barrier integrity ↑ , TNF-α, RANKL, IL-17 mRNA expression ↓	^30^
	*L. rhamnosus* GG	Conventionally raised C57BL/6 mice	Femur BV/TV, Spine BV/TV ↑ , serum CTX ↓	MAR, P1NP, Wnt10b expression in BM and CD8^+^ T cells, Foxp3^+^ T cells in BM, spleen, and PP ↑	^33^
	*L. acidophilus* ATCC 4356	OVX Balb/c mice	Bone mass ↑ , Femur resorption pits/lacunae ↓	CD4^+^ Foxp3^+^ Treg cells, CD8^+^ Foxp3^+^ Treg cells, serum IL-10, IFN-γ ↑ , CD4^+^Rorγt^+^ Th17 cells, serum IL-17, TNF-α, RANKL ↓	^174^
	*L. sakei*	RA DBA/1 J mice	Arthritis score, TRAP^+^-, RANK^+^-, RANKL^+^ -cells ↓	TNF-α, IL-1β, IL-6, IL-17 ↓	^180^
	*L. fermentum* ZS40	OP Wistar rats	BV/TV, Tb. N., Tb. Th., BMD ↑, the number of OC, Tb spacing ↓	β-catenin, Wnt10b, Lrp5, Lrp6, Runx2, ALP, RANKL, OPG mRNA expression ↑ , DKK1, RANK, TRAP, CTSK mRNA expression ↓	^175^
	*L. Plantarum* HFY15	OP Wistar rats	BV/TV, Tb. N., Tb. Th., BMD ↑, the number of OC, Tb spacing ↓	β-Catenin, Wnt10b, Lrp5, Lrp6, Runx2, ALP mRNA expression ↑ , DKK1, RANK, TRAP, CTSK mRNA expression ↓	^176^
	*L. helveticus* ATCC 27558	OVX Sprague–Dawley rats	Femur BMD, breaking forces, serum OCN ↑ , serum CTX ↓	Runx2 and BMP2 mRNA expression ↑ , *Lactobacillus* enumeration in the feces ↑	^177^
	*L. curvatus* Wikim 38	OVX C57BL/6 mice	BV/TV, BMD, Tb. Th. ↑	F-actin ring formation, bone resorption, osteoclastogenesis, RANKL/TRAF6/ NF-κB/MAPK gene expression ↓	^178^
	*L. paracasei* DSM13434, or a mixture of *L. paracasei* DSM13434, *L. plantarum* DSM 15312 and DSM 15313	OVX C57BL/6 N mice	Cb. BMC., Cb. Area ↑	BM TNF-α, IL-1β, OPG ↑	^179^
	*L. reuteri* ATCC PTA 6475	Postmenopausal women with osteopenia	The loss of total BMD ↓		^189^
	*L. casei Shirota*	Elderly patients with an acute distal radius fracture	DASH (disabilities of the arm, shoulder, and hand) score, pain, complex regional pain syndrome score, wrist flexion, and grip strength of patients receiving probiotics exhibited a significantly faster pace of improvement.		^190^
	*L. paracasei* DSM 13434, DSM 15312, and DSM 15313	Postmenopausal women with osteopenia	Lumbar spine bone loss ↓		^188^
*Bifidobacterium*	*B. longum*	OVX Sprague-Dawley rats	Serum OCN, femur BMD, Tb. Th., femur strength ↑ , Serum CTX ↓	Sparc and BMP2 mRNA expression ↑ , Total *Bifidobacteria* in the feces ↑	^184^
	*B. longum* 35624	OC precursors from C57BL/6 N mice	OC differentiation ↓	Surface exopolysaccharide of *B. longum* 35624 mediated inhibition of osteoclast formation is TLR2-dependent.	^186^
	*B. adolescentis*	Fractured C57BL/6 mice	Serum P1NP, callus cartilage remodeling ↑ , post-traumatic bone loss ↓	Tight junction protein ↑ , Systemic inflammation ↓	^187^
	*B. pseudocatenulatum* CECT 7765	Obesity C57BL/6 mice	Tb. N., Serum OCN ↑ , Tb spacing, Serum CTX ↓ ;	Canonical Wnt/β-catenin pathway ↑	^185^
	VSL#3 (*B. breve*, *B. longum*, *B. infantis*, *L. acidophilus*, *L. plantarum*, *L. paracasei*, *L. bulgaricus*, *and Streptococcus thermophilus*)	OP C57BL6/J mice induced by leuprolide administration	Femur BV/TV, Cb. V., serum OCN ↑	Intestinal tight junction ↑	^30^
*Akkermansia*	*A. muciniphila*	OVX C57BL/6 mice	BMD, Tb. BV/TV., Tb. N., serum OCN, MAR ↑ , Tb spacing, the number and size of OC ↓	EVs are required for the *A. muciniphila*‐induced bone protective effects.	^159^
	*A. muciniphila*	C57/BL6 mice with experimental periodontitis	Alveolar bone loss ↓	M2 macrophages, IL-10 gene expression ↑	^201^
	*A. muciniphila*	Fractured C57BL/6 mice	BV/TV, tissue mineral density of callus, femoral ultimate load, serum OCN, bone fracture healing ↑	Type H vessel formation in callus ↑, inflammatory responses in fracture healing, intestinal permeability, and inflammation ↓	^203^

↑: up-regulated or increased compared to those mice without treatment

↓: down-regulated or decreased compared to those mice without treatment

*ALP* Alkaline phosphatase, *BM* Bone marrow, *BMC* Bone mineral content, *BMD* Bone mineral density, *BMP2* Bone morphogenetic protein 2, *BV/TV* The ratio of bone volume to total volume, *Cb* Cortical bone, *CTSK* Cathepsin K, *CTX-I* C-terminal telopeptides of type I collagen, *DKK1* Dickkopf-related protein 1, *EVs* Extracellular vesicles, *IFN-γ* Interferon-γ, *IL* Interleukin, *LRP* Low-density lipoprotein receptor-related protein, *MAPK* Mitogen-activated protein kinase, *MAR* Mineral apposition rate, *OB* Osteoblasts, *OC* Osteoclasts, *OCN* Osteocalcin, *OP* Osteoporosis, *OPG* Osteoprotegerin, *OVX* Ovariectomized, *P1NP* Procollagen type I N-terminal propeptide, *PPs* Peyer’s patches, *RA* Rheumatoid arthritis, *RANKL* Receptor activator of nuclear factor kappa-B ligand, *Tb. N.* Trabecular bone number, *Tb. Th.* Trabecular bone thickness, *Tb* Trabecular bone, *TGF-β* Transforming growth factor-β, *TLR* Toll-like receptor, *TNF-α* Tumor necrosis factor-α, *TRAF6* Tumor necrosis factor receptor associated factor 6, *TRAP* Tartrate-resistant acid phosphatase, *WT* Wild-type

### Conventional probiotics: *Lactobacillus* and *Bifidobacterium*

As a conventional probiotic, *Lactobacillus* has a long history of use.^162^ It is associated with various health benefits, including relief from diarrhea, irritable bowel syndrome (IBS), IBD, lactose intolerance, and obesity.^163–167^ *Lactobacillus*-fermented products, such as milk, soy skim milk, and kefir (fermented milk similar to yogurt with a history of over one hundred years), have been reported to have a beneficial effect on bone health.^168–172^ *Lactobacillus* supplementation has been shown to prevent bone loss induced by OVX,^25,30,34,173–179^ reduce the number of TRAP^+^ cells, receptor activator of nuclear factor kappa-B (RANK)^+^ cells, and RANKL^+^ cells in rheumatoid arthritis (RA) mice,^180^ and improve skeletal health in intact animals.^33,181^ *Bifidobacterium*, on the other hand, was markedly reduced in the senescence-induced OP model.^182^ Mice fed a low-calcium diet also had lower BMD and a decreased abundance of *Bifidobacterium*.^183^ *Bifidobacterium* has been proven to prevent bone loss induced by OVX or obesity,^184,185^ inhibit pre-OC differentiation in vitro,^186^ and accelerate callus cartilage remodeling in fractured mice.^187^ Administration of a probiotic cocktail (VSL#3 or a mixture of *Lactobacillus paracasei (L. paracasei)* DSM 13434, *L. plantarum* DSM 15312, and DSM 15313) could increase femur BV/TV and Cb. BMC in OVX mice.^30,179^ The mixture of *L. paracasei* DSM 13434, DSM 15312, and DSM 15313 has been used in clinical trials and proven to prevent lumbar spine bone loss in postmenopausal women with OP.^188^ Treatment with *L. reuteri* ATCC PTA 6475 or *Lactobacillus casei Shirota* (*L. casei Shirota*) alone could reduce BMD or accelerate distal radius fracture healing in the elderly.^189,190^

In addition, *Lactobacillus* and *Bifidobacterium* have been engineered as delivery vectors for specific molecules.^191,192^ The premise is that such bacterial vectors do not produce any virulence factors and are tolerated by the host.^162^ For example, *Lactococcus lactis* (*L. lactis*) was engineered to deliver IL-10 to control allergen sensitivity.^193^ Oral administration of *L. lactis*, engineered to express and deliver elafin (a natural protease inhibitor with pleiotropic anti-inflammatory properties), could decrease inflammation and restore intestinal homeostasis in mouse models of acute and chronic colitis.^191^ Another human commensal bacterium, *Bacteroides ovatus*, was also engineered for in situ delivery of TGF-β and treatment of colitis.^192^ Further studies are needed to explore the role of such engineered bacteria in bone metabolism.

### Next-generation probiotics

The development of new probiotics, also known as “next-generation probiotics,” has gradually gained traction recently.^162^ One such probiotic is *A. muciniphila*, a newly identified genus in the phylum Verrucomicrobia that is a symbiotic bacterium in the mucus layer and utilizes mucus as a single nutrient source.^116,194^ Given its safety and pivotal roles in alleviating obesity and IBD, *A. muciniphila* is widely considered a next-generation probiotic.^194–200^

Research has shown that *A. muciniphila* is abundant in children’s GM, which may explain why FMT from children provides better protection against OVX-induced bone loss compared to GM transplantation from older people.^159^ *A. muciniphila* is also correlated with bone physiology, and there is a direct correlation to bone formation. For example, Chevalier et al. reported that warm temperature exposure (34 °C), rather than room temperature (RT) conditions, improved the tibial BV/TV and the abundance of *Akkermansia* in 24-week-old female mice.^8^ In contrast, OVX mice had a lower abundance of *A. muciniphila* than mice with sham operations (Sham).^159^ Warm-exposed-FMT increased tibial breaking strength and BV in OVX mice compared with RT-exposed-FMT.^8^ *A. muciniphila* has also been reported to promote the healing of bone fractures and mitigate *Porphyromonas gingivalis*-induced alveolar bone destruction.^201–203^ Accumulated research has revealed that both live and pasteurized *A. muciniphila* could prevent or treat obesity-related metabolic disorders and systemic inflammation.^194–199,204–208^ However, pasteurized *A. muciniphila* provides no protection against OVX-induced bone loss.^209^

Despite the growing interest in the use of next-generation probiotics as a potential therapy for OP, research in this field remains in its infancy. Many novel probiotics, which were discussed in the review by O’Toole et al., have not been used in bone research.^162^ For instance, *Clostridium butyricum* (*C. butyricum*) is a strain of the *Clostridium* genus found in various environments, including soil, cultured milk products, vegetables, and the human colon.^210^ *C. butyricum* can utilize undigested dietary fibers and generate SCFAs, specifically butyrate and acetate.^121^ *C. butyricum* MIYAIRI 588 (CBM 588) is widely used as a novel food ingredient or a treatment for diarrhea due to its safe, nonpathogenic, and nontoxic profile.^210–212^ Given the benefits of butyrate on bone formation, we suggest that CBM 588 or other SCFA-producing GM can be applied to research on the gut-bone axis. *Faecalibacterium prausnitzii* (*F. prausnitzii*) is a major member of Firmicutes phylum. The reduction in *F. prausnitzii* is associated with a higher risk of postoperative recurrence of ileal CD. The metabolites of *F. prausnitzii* suppress NF-κB activation and IL-8 production.^213,214^ *F. prausnitzii* increased plasma anti-Th17 cytokines (IL-10 and IL-12) and decreased plasma IL-17 levels in colorectal colitis rats. The culture supernatant of *F. prausnitzii* also suppressed Th17 cell differentiation in vitro.^215^ Given that immune cytokines regulate bone metabolism, it is necessary to explore the effect of these anti-inflammatory probiotics in protecting against bone loss.

### Problems with probiotic screening

Khosla and Hofbauer conducted a comprehensive review of the advancements and challenges in OP treatment and indicated that the progression of recent OP drugs and new pharmacological approaches will probably be based on mechanisms of rare diseases and fundamental bone biology.^9^ The development of these new medications provides a basis for the exploration of probiotic treatments. The prolonged use of anti-resorption drugs, however, is associated with certain risks. For example, combined hormone therapy increases the risk of cardiovascular disease in patients over 70 years old, denosumab is associated with the risk of osteonecrosis of the jaw and atypical femur fractures, and bisphosphonates have been shown to suppress bone turnover markers for at least five years after discontinuation.^2,9^ Apart from these limitations, most drugs, except bisphosphonates and strontium ranelate, have failed to sustain their bone-anabolic effects following discontinuation.^9^ Probiotic treatments are expected to address such drawbacks and provide sustained benefits in terms of bone mass, provided they are effectively colonized in the host body. Nonetheless, further research is necessary to fully comprehend the mechanisms of host colonization and the long-term effects of such colonization.

A decreased diversity in the GM is regarded as an indicator of various pathological conditions, such as inflammatory and metabolic disorders.^216,217^ The causal relationship between GM composition and OP remains controversial. Huang et al. conducted an analysis of 12 prior studies to examine the differences in GM abundance between OP patients and healthy individuals, which included fecal GM data from 2 033 people (604 with OP and 1 429 healthy controls).^218^ The findings revealed that the relative abundance of *Lactobacillus* and *Ruminococcus* increased in the OP group, while the relative abundance of *Bacteroides* in the Bacteroidetes phylum increased (with the exception of Ireland). Conversely, the relative abundance of the genera *Blautia*, *Alistipes*, *Megamonas*, and *Anaerostipes* decreased in Chinese OP patients.^218^ However, as mentioned by the author, most of the included studies exhibited significant heterogeneity, and individual differences, such as sample size, race, residence, diet, medication, age, gender, physical exercise, and stress levels, often impacted GM composition. As a result, it remains uncertain whether changes in GM composition can serve as a biomarker for OP. Nonetheless, these findings provide valuable insights for the selection of probiotics. For instance, *Blautia* is a genus of anaerobic bacteria that can generate SCFAs from dietary fiber and modulate the immune response of Treg cells.^219,220^ *Alistipes*, which belongs to the Bacteroidetes phylum, was reported to generate acetate and propionate.^221,222^ *Alistipes finegoldii* is suggested to be protective against colitis.^223^ *Anaerostipes* was suggested to have potential benefits due to its ability to produce SCFAs.^224,225^ It is important to determine the changes in GM composition, particularly with regards to bacteria that have potential benefits, following probiotic administration. For instance, the relative abundance of phylum *Verrucomicrobia* and genus *Akkermansia* was decreased in OP mice,^25,159^ but transplantation of GM rich in *A. muciniphila* or administration of LGG could restore their abundance.^25,159^ Administration of LGG has also been reported to alter microbial diversity and increase the proportion of SCFA-producing Clostridia in conventionally raised mice.^33^ Furthermore, feeding rats *Lactobacillus helveticus* was associated with a significant increase in the number of *Lactobacillus* colonies in their feces compared to the sham and OVX groups.^177^

Studies that assess the impact of probiotics on bone health have primarily used OVX animals as experimental models. It is important to consider not only preventing adverse health outcomes but also promoting positive ones, especially increasing bone mass during skeletal maturity (peak BMD). Maintaining peak BMD is crucial for good bone health, as a 10% increase in peak BMD has been estimated to delay the onset of OP by 13 years.^226^

## Conclusion

Research on the GM in the gut–bone axis has yielded novel insights into the pathogenesis of OP and the potential for using gut microbes as a treatment strategy. However, the inconsistency of test environments, the host’s genetic backgrounds, and the sources of gut microbes pose significant challenges in controlling variables in research. Furthermore, the response of the host GM to FMT or probiotic stimulation could potentially interfere with the colonization of exogenous gut microbes. Therefore, the transition from basic research to clinical research and the practical application of probiotics remains a challenge. It is imperative to continue the search for effective probiotics for OP treatment and to meticulously evaluate their quality, safety, dosage, stability, and interactions with other medications.
